# Trends in opioid dispensing to injured workers following codeine scheduling changes in Australia: a retrospective cohort study

**DOI:** 10.1136/bmjopen-2024-092651

**Published:** 2025-03-12

**Authors:** Michael F Di Donato, Stephanie Mathieson, Giovanni E Ferreira, Ting Xia, Yonas Getaye Tefera, Christina Abdel Shaheed, Christopher Maher, Alex Collie

**Affiliations:** 1 School of Public Health and Preventive Medicine, Monash University, Melbourne, Victoria, Australia; 2 Sydney Musculoskeletal Health, The University of Sydney, Sydney, New South Wales, Australia; 3 Institute for Musculoskeletal Health, Sydney Local Health District, Camperdown, New South Wales, Australia; 4 Monash Addiction Research Centre, Monash University, Melbourne, Victoria, Australia

**Keywords:** Retrospective Studies, OCCUPATIONAL & INDUSTRIAL MEDICINE, Health policy, Back pain

## Abstract

**Abstract:**

**Objectives:**

To describe the prevalence and patterns of opioid analgesic and pain medicine dispenses, and the impact of up-scheduling of low-dose (≤15 mg) codeine-containing products to Australians with accepted workers’ compensation time loss claims for musculoskeletal conditions between 2010 and 2019.

**Design:**

Interrupted time series.

**Setting:**

Workers’ compensation scheme in Victoria, Australia.

**Population:**

Australians with accepted workers’ compensation time loss claims for musculoskeletal conditions between 2010 and 2019.

**Main outcome measures:**

Number and proportion of workers dispensed pain medicines in the first year of claim and the monthly number, percentage of pain medicine dispenses and mean morphine equivalent dispense dose.

**Results:**

Nearly one-third (28.4%, n=22 807) of our sample of 80 324 workers were dispensed any opioid in the first year since the workers’ compensation insurer received their claim. There were no significant step or trend changes in the number or percentage of pain medicines dispensed of up-scheduled low-dose codeine. Only 2.9% of workers were ever dispensed up-scheduled low-dose codeine, specifically 2.5% after up-scheduling (1 February 2018). After up-scheduling of low-dose codeine, workers were more likely to be dispensed opioids (excluding codeine) (prevalence ratio (PR) 1.21, 99% CI 1.13, 1.31) or other pain medicines (eg, pregabalin, paracetamol) (PR 1.11, 99% CI 1.03, 1.19) compared with the year prior. There was a significant 28.5% (99% CI 16.3, 41.9) step increase (ie, increase immediately after up-scheduling) in high-dose (>15 mg) codeine with a significant trend decrease (−1.3%, 99% CI −2.5, –0.2).

**Conclusion:**

Up-scheduling low-dose codeine to prescription-only medicines did not significantly change the dispensing of low-dose codeine-containing products to workers with accepted workers’ compensation time loss claims for musculoskeletal conditions.

**STRENGTHS AND LIMITATIONS OF THIS STUDY**

- Our study used a large sample of detailed claims and medicine data enabling us to gain new insights about a critical policy change.
- This is the first study to report on the impact of codeine scheduling changes in the compensable patient population.
- Our study is limited by background trends in medicine use, a relatively short 12-month follow-up period, and only including medicines funded by the workers’ compensation scheme.

## Introduction

 Recommendations for the use of opioid analgesics have changed in recent years with greater recognition that the risks may outweigh the potential benefits.^1 2^ These side effects and the risk of opioid overuse and overdose have become particularly pertinent issues for policymakers globally. In Australia, this has meant the introduction of real-time prescription drug monitoring programmes^3^ and restrictions on the access to some opioids. Low-dose codeine-containing products (ie, ≤15 mg of codeine per dose unit) have been progressively restricted to reduce harmful use over several years.^4^ Originally available at pharmacies and licensed retailers in Australia, supply was first restricted to over-the-counter purchases (ie, available without a prescription) only at pharmacies in 2010.^5^ A major change was implemented on 1 February 2018, when low-dose codeine products were ‘up-scheduled’ from Schedule 3 to Schedule 4, restricting access to prescription-only.

Musculoskeletal conditions and injuries are the leading cause of workers’ compensation claims in Australia, accounting for half (50.3%, n=64 300) of time loss claims in 2021–2022.^6^ Workers’ compensation schemes fund reasonable and necessary healthcare, including medicines.^7^ Costs for over-the-counter medicines may be reimbursed to the worker directly. However, prescription medicines require a consultation with a medical practitioner to first obtain a prescription. While workers’ compensation funds this consultation, it is an additional step that may have impacted opioid-seeking behaviour in injured workers.

Several studies have highlighted the impact of up-scheduling low-dose codeine on codeine supply, other opioid supply,^8 9^ overdoses,^10^ opioid use disorders^11^ and emergency department presentations.^12^ However, these studies were in the general population. Work-related injuries present a unique set of factors that affect recovery and a funding mechanism for healthcare and medicines that differs from the mainstream universal public health model in Australia.^13 14^ Therefore, we sought to (1) describe the prevalence and patterns of opioid and pain medicine dispensing over time and (2) examine the association between the up-scheduling of low-dose codeine and the number and proportion of opioids and pain medicines, and the dose of opioids, dispensed to injured workers with musculoskeletal conditions.

## Methods

### Setting

Workers’ compensation schemes in each Australian state and territory fund income replacement and healthcare for workers where injury can be attributed to employment.^7^ Approximately 90% of workers in the state of Victoria, Australia, are covered by workers’ compensation insurance. Victoria is Australia’s second most populous state, with a labour force of 3.2 million people when low-dose codeine was up-scheduled in 2018.^15^ The Victorian workers’ compensation scheme requires that employers fund the first 10 business days of income replacement and $700 (AUD) of medical expenses.^16^

### Data source

We used a sample of the workers’ compensation claims data provided by the Victorian workers’ compensation scheme regulator, WorkSafe Victoria.^17^ Data contained information about the worker (ie, sex, age, occupation, injury details, employer size and type, key dates) and detailed medicine dispense data (ie, medicine type and ingredients, dispense date, dispense dose, cost). A medicine dispense was considered the total number of units (eg, tablets) dispensed on a given date, typically a single packet of medicine, for example, 20 tablets of codeine and paracetamol. Preparing claim data for analysis involved quality assurance of several variables (eg, age, sex, occupation) and joining population socioeconomic (Index of Relative Socio-economic Advantage and Disadvantage (IRSAD)) and remoteness (Accessibility/Remoteness Index of Australia (ARIA)) measures by worker residential postcode.^18 19^ Cleaning medicine data involved applying the Anatomical Therapeutic Chemical (ATC) coding scheme^20^ and checking variables used in the calculation of opioid dose, morphine milligram equivalent dose and the calculation of opioid dose itself (ie, dispense quantity * item strength * morphine multiplier). Access to data was approved by the Monash University Human Research Ethics Committee (Project ID 30718).

### Sample

We included workers with accepted workers’ compensation claims for musculoskeletal conditions received by insurers between 1 February 2010 and 31 January 2019. Musculoskeletal conditions were defined by the Type of Occurrence Classification System (TOOCS; online supplemental table 1).^21^ Only workers with at least 1 day of income replaced by the workers’ compensation scheme were included. Eligible workers were aged between 15 and 80 years.

We included pain medicines defined by ATC level 2 codes relating to the musculoskeletal and nervous systems: M01, M02, M03, N01, N02, N03, N05 and N06 (online supplemental table 2). Included medicines were classified as either up-scheduled low-dose codeine (≤15 mg), high-dose codeine (>15 mg), opioids (excluding codeine) or other pain medicines using ATC codes and item strength (see online supplemental table 3). We included any opioid or other pain medicine dispensed to eligible workers 31 days before to 365 days after the date the insurer received the workers’ compensation claim (ie, their first year of claim). Although rare, healthcare funded by workers’ compensation may be retrospectively reimbursed, hence the 31 days prior. Our follow-up data allowed for a 1-year (ie, 365 day) follow-up period. Opioid dispenses with missing dose information or illogical dose quantities (eg, >240 tablets for codeine and paracetamol) were excluded from analyses. This accounted for approximately 0.59% of all opioid dispenses and 0.21% of opioid dispenses included in final interrupted time-series models (online supplemental table 4).

### Outcome variables

Changes in the prevalence of any pain medicines dispensed were measured by the number and proportion of workers dispensed each type of pain medicine in the year since an insurer received their claim. Changes in monthly pain medicine dispenses were measured by the number of each type of pain medicine, the percentage of pain medicines dispensed and mean morphine equivalent (MME) dispense dose (for opioids).

### Analysis

We first grouped workers into 1 year intervals that aligned with the up-scheduling of low-dose codeine (1 February 2018) by the date the insurer received their claim. That is, each year commenced on 1 February and ended 31 January the following year. For example, if a worker’s claim was received by the insurer on 2 January 2013, they would be assigned as a claim commencing in 2012. We used descriptive statistics to report the number and proportion of workers per year who were dispensed each category of pain medicine (online supplemental table 2) at any time in the first year of their claim. We then used Poisson models to compare the likelihood of being dispensed each of those pain medicines relative to the year prior to up-scheduling (1 February 2017 to 31 January 2018). This allowed us to compare the prevalence of pain medicines in the year after up-scheduling to the year prior, but also to previous years in the sample. Poisson models adjusted for worker sex, age group (ie, 15–24, 25–34, 35–44, 45–54, 55–64 and 65 or more years), employment type (ie, full-time, part-time, casual or other), employer size (ie, government, large, medium or small), nature of injury (TOOCS),^21^ bodily location of injury (TOOCS), occupation (Australian Standard Classification of Occupations major groups),^22^ socioeconomic status (IRSAD quintiles)^18^ and remoteness (ARIA).^19^ We used the log of the total number of workers as offsets and robust standard errors in each model. Results were reported as prevalence ratios (PR) with 99% CI (PR 99% CI) and considered statistically significant where p<0.01.

We then visualised the monthly percentages of (1) pain medicines by pain medicine category and (2) the five most frequently dispensed opioids, both over the entire time series (ie, 2010 to 2019).

We used interrupted time-series analyses to measure the impact of the up-scheduling of low-dose codeine on pain medicine dispensing. We selected a time series of medicines dispensed 2 years before to 1 year after low-dose codeine was up-scheduled (ie, dispensed between 1 February 2016 and 31 January 2019). In total, 36 time points were included in the analyses. We used descriptive statistics to report the monthly mean and SD of the number, percentage of pain medicines dispensed and mean dispensed dose of each category of pain medicine. Negative binomial models were used to measure changes in the number of dispenses. The output of negative binomial models was converted to a percentage change (ie, 100 * incidence rate ratio − 100). Generalised least squares (GLS) models were used to measure changes in the rate and mean dose of dispenses. We log-transformed the response variable in GLS models (ie, rate and dispense dose) to make the output a percentage change. We tested for seasonality in all models by adding six sine and six cosine terms to the initial models. Seasonality terms that were significant (p<0.05) were retained in the final models. Autoregressive moving average methods were used to assess and adjust models for autocorrelation and partial autocorrelation.^23^ Akaike and Bayesian Information Criteria were used to compare and select the final models. We considered results as statistically significant where p<0.01. We reported results in percentage change in step (ie, immediate change in outcome following the month of up-scheduling) and trend (ie, a change in trend slope following the month of up-scheduling) with 99% CI.^23 24^ Results were visualised with time-series figures reporting the original data, trends before and after the up-scheduling of low-dose codeine and the counterfactual trend after up-scheduling (ie, trend if up-scheduling had not occurred). We performed analyses in RStudio using R 4.2.2 and several R packages (online supplemental table 5).

### Patient and public involvement

Patients and/or the public were not involved in the design, conduct, reporting or dissemination plans of this research, due to the nature of the de-identified data. We sought expert input on the final manuscript from the workers’ compensation regulator, WorkSafe Victoria, prior to submission.

## Results

Our sample included 80 324 workers. Nearly one-third (28.4%, n=22 807) were dispensed any pharmaceutical opioid analgesic and 25.9% (n=20 790) other medicines to manage pain conditions in the first year since the insurer received their claim (see table 1). Specifically, 2.9% (n=2367) were dispensed up-scheduled low-dose codeine, 12.9% (n=10 358) high-dose codeine and 22.6% (n=18 154) opioids (excluding codeine) (see table 2). The proportion of workers who were dispensed each medicine by all available covariates is available in the supplementary materials (online supplemental table 6).

**Table 1:** Statistical comparison of the prevalence of all opioids and other pain medicines by the year insurers received workers’ claims

Year the insurer received the claim*	Workers	All opioids (incl. codeine)		Other pain medicines	
	N	N (%)	PR (99% CI)†‡	N (%)	PR (99% CI)†‡
2010	9141	2751 (30.1)	1.13 (1.06, 1.20)§	2426 (26.5)	1.05 (0.98, 1.13)
2011	9011	2616 (29.0)	1.09 (1.02, 1.16)§	2390 (26.5)	1.06 (0.99, 1.13)
2012	9400	2644 (28.1)	1.04 (0.98, 1.11)	2356 (25.1)	0.99 (0.93, 1.07)
2013	8835	2433 (27.5)	1.03 (0.97, 1.10)	2274 (25.7)	1.02 (0.96, 1.10)
2014	8873	2475 (27.9)	1.04 (0.98, 1.11)	2310 (26.0)	1.05 (0.98, 1.12)
2015	8644	2457 (28.4)	1.07 (1.00, 1.14)	2309 (26.7)	1.08 (1.01, 1.16)
2016	8680	2347 (27.0)	1.01 (0.94, 1.08)	2202 (25.4)	1.02 (0.95, 1.10)
2017	8749	2310 (26.4)	1.00 (ref)	2113 (24.2)	1.00 (ref)
2018	8991	2774 (30.9)	1.18 (1.11, 1.26)§	2410 (26.8)	1.11 (1.03, 1.19)§
All years	80 324	22 807 (28.4)	–	20 790 (25.9)	–

* Year that the insurer received the claim, where each year commenced on 1 February and ended on 31 January.

† Prevalence ratio and 99% CI.

‡ Poisson model adjusted for worker sex, age group, employment type, employer size, nature of injury, bodily location of injury, occupation, socioeconomic status and remoteness. Full models available in supplementary materials (online supplemental table 7).

§ p<0.01.

PRprevalence ratio

**Table 2:** Statistical comparison of the prevalence of each type of opioid by the year insurers received workers' claims

Year the insurer received the claim*	Workers	Up-scheduled low-dose codeine		High-dose codeine		Opioids (excl. codeine)	
	N	N (%)	PR (99% CI)†‡	N (%)	PR (99% CI)†‡	N (%)	PR (99% CI)†‡
2010	9141	359 (3.9)	1.52 (1.21, 1.92)§	1530 (16.7)	1.75 (1.57, 1.94)§	1999 (21.9)	0.97 (0.90, 1.05)
2011	9011	340 (3.8)	1.48 (1.17, 1.87)§	1382 (15.3)	1.61 (1.44, 1.80)§	1996 (22.2)	0.98 (0.91, 1.06)
2012	9400	313 (3.3)	1.27 (1.00, 1.61)	1370 (14.6)	1.52 (1.36, 1.69)§	1978 (21.0)	0.92 (0.86, 1.00)
2013	8835	263 (3.0)	1.16 (0.91, 1.49)	1197 (13.5)	1.42 (1.27, 1.60)§	1887 (21.4)	0.94 (0.88, 1.02)
2014	8873	210 (2.4)	0.93 (0.72, 1.21)	1194 (13.5)	1.41 (1.26, 1.58)§	1964 (22.1)	0.98 (0.91, 1.06)
2015	8644	222 (2.6)	1.00 (0.78, 1.30)	1053 (12.2)	1.29 (1.14, 1.45)§	1996 (23.1)	1.03 (0.96, 1.11)
2016	8680	228 (2.6)	1.05 (0.82, 1.36)	909 (10.5)	1.10 (0.97, 1.24)	1984 (22.9)	1.01 (0.94, 1.09)
2017	8749	210 (2.4)	1.00 (ref)	815 (9.3)	1.00 (ref)	1947 (22.3)	1.00 (ref)
2018	8991	222 (2.5)	1.05 (0.82, 1.36)	908 (10.1)	1.09 (0.96, 1.23)	2403 (26.7)	1.21 (1.13, 1.31)§
All years	80 324	2367 (2.9)	–	10 358 (12.9)	–	18 154 (22.6)	–

* Year that the insurer received the claim, where each year commenced on 1 February and ended on 31 January.

† Prevalence ratio and 99% CI.

‡ Poisson model adjusted for worker sex, age group, employment type, employer size, nature of injury, bodily location of injury, occupation, socioeconomic status and remoteness. Full models available in supplementary materials.

§ p<0.01.

PRprevalence ratio

### Changes in the prevalence of pain medicines

Opioids (including codeine) and other pain medicines were significantly more prevalent in workers whose claims began in the year after the up-scheduling of low-dose codeine relative to the year prior (see table 1). The lowest proportion of workers dispensed opioids was observed in the year prior to the up-scheduling of codeine. Opioids were significantly more prevalent in workers whose claims began in 2010 and 2011. There were no significant differences in the prevalence of other pain medicines in any year prior to 2017.

There were no significant differences in the prevalence of up-scheduled low-dose codeine or high-dose codeine in the year after low-dose codeine was up-scheduled. The greatest number and proportion of workers dispensed up-scheduled low-dose codeine and high-dose codeine were in workers whose claims were received by the insurer in 2010 at 3.9% and 16.7%, respectively (see table 2). High-dose codeine was dispensed to a significantly greater percentage of workers with claims commencing between 2010 and 2015 compared with the reference year (2017). There were no significant differences in the prevalence of opioids (excluding codeine) over the study period, except for those workers whose claims commenced after the up-scheduling of low-dose codeine (PR 1.21, 99% CI 1.13, 1.31). The prevalence of workers whose claims were received by the insurer in this year increased to 26.7% from 22.3% in the previous year. Full models are available in supplementary materials (online supplemental table 7).

### Changes in pain medicine dispensing

**Figure 1:**
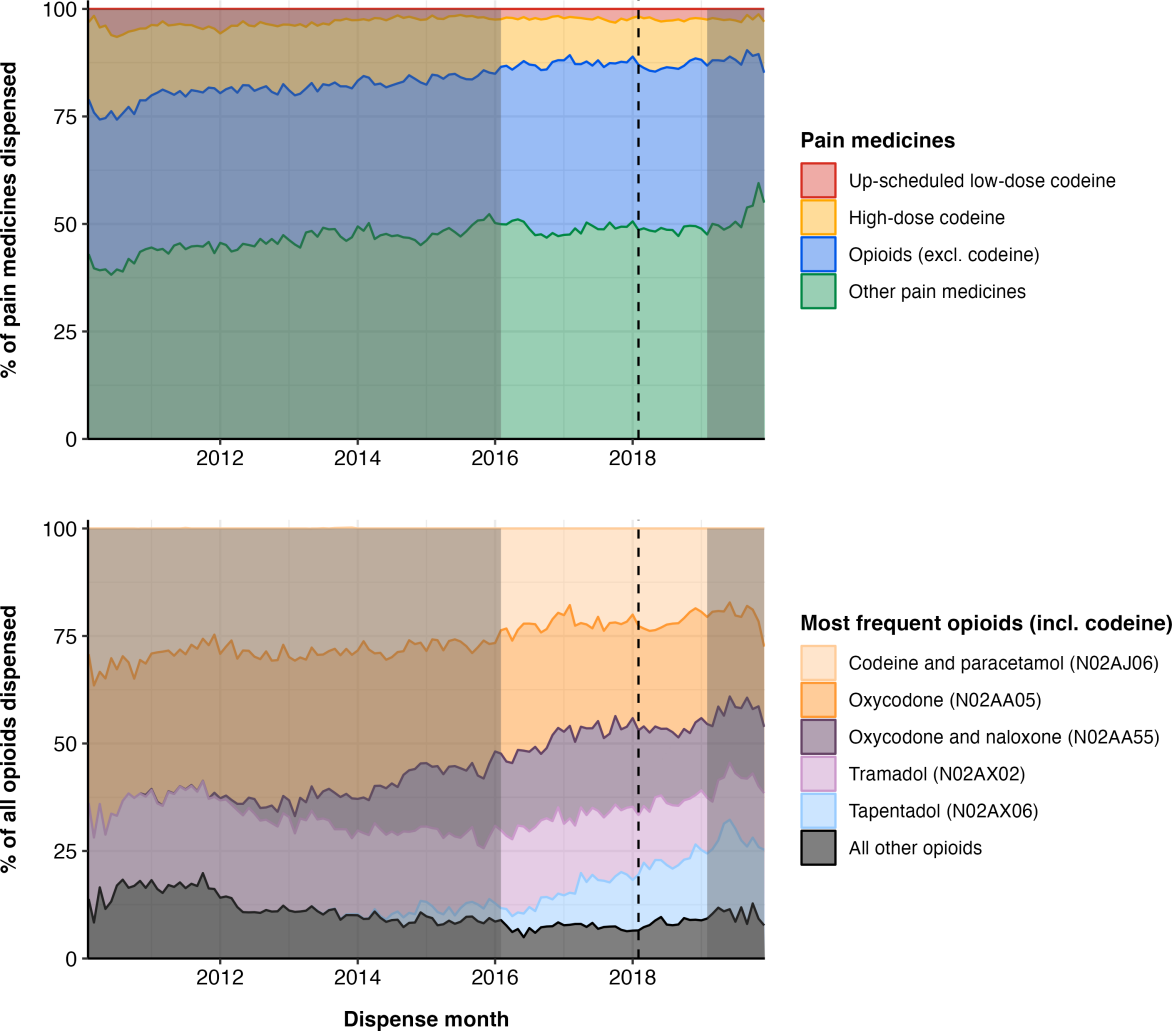
Trends in pain medicines and most frequent opioids throughout the sample period. Dotted line indicates up-scheduling of codeine; highlighted period indicates time series (ie, 2016 to 2019) used in interrupted time-series analyses.

Opioid analgesics were a common pain medicine dispensed throughout the sample period (figure 1). Up-scheduled low-dose codeine contributed to a consistently small proportion of pain medicines dispensed. High-dose codeine products appeared to decrease over the 10 year sample period.

The five most frequent opioids dispensed fluctuated over the study period. Single-ingredient oxycodone and codeine in combination with paracetamol were the most frequent opioids throughout the sample period. A complete list of the most frequent pain medicines and pain medicine strengths is available in the supplementary materials (online supplemental table 8). Figure 1 also highlights the up-scheduling of low-dose codeine in February 2018 (dotted line) and 3 years of data used in the interrupted time series (highlighted segment).

**Figure 2:**
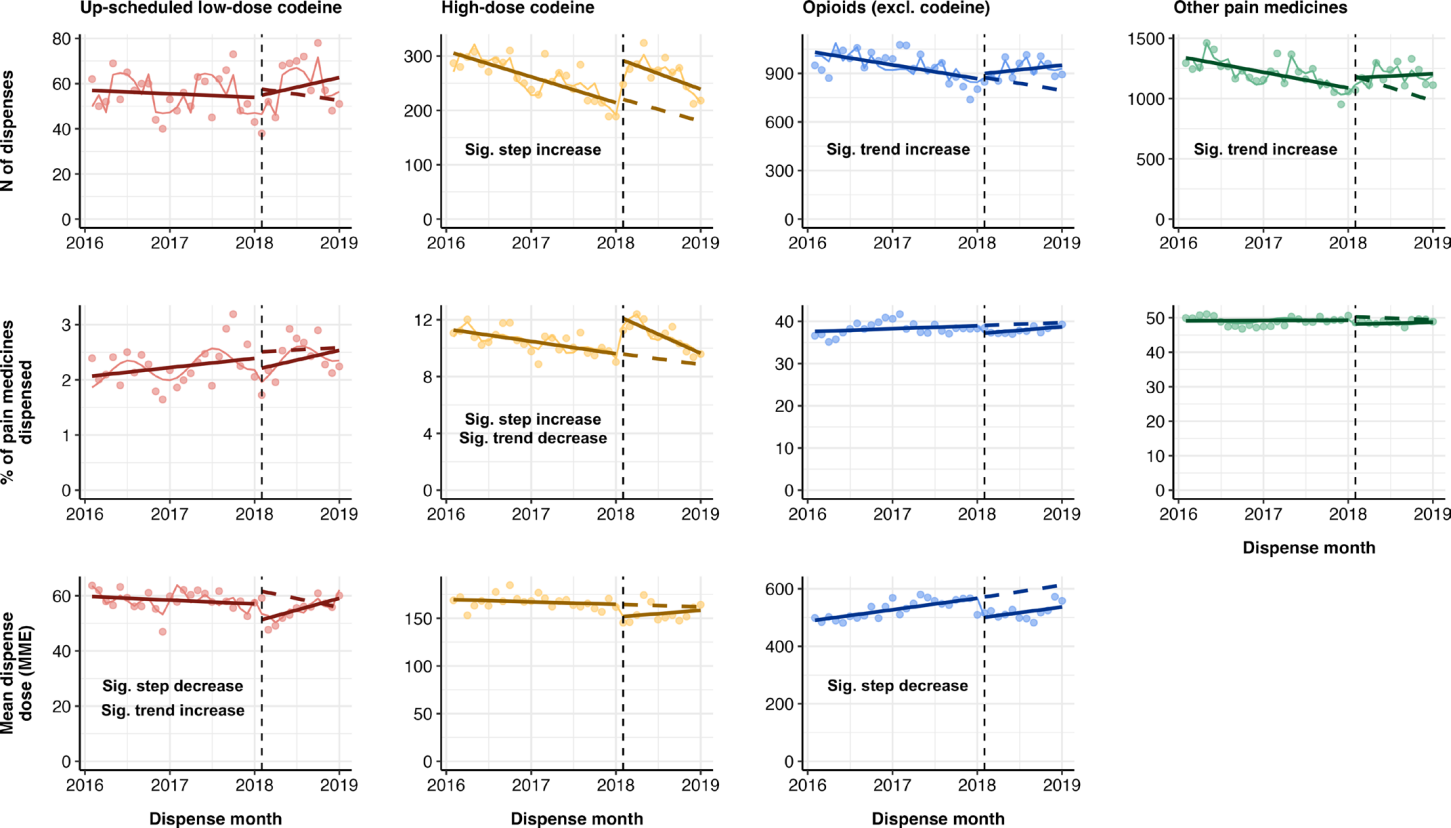
Results of interrupted time series. Points = original data; solid line = trend; dotted line = counterfactual trend.

There were no significant step or trend changes in the number of up-scheduled low-dose codeine dispenses or the percentage of pain medicines that were up-scheduled low-dose codeine (see table 3 and figure 2). However, there was a significant 18.5% (99% CI –27.7, –12.7%) step decrease in mean dispense dose at the month of up-scheduling implementation, but with a significant trend increase of 2.2% per month following the up-scheduling implementation (99% CI 0.8%, 3.6%). We observed a significant 32.3% (99% CI 17.4%, 49.0%) step increase in the number of high-dose codeine dispenses following up-scheduling of low-dose codeine. This was accompanied by a 28.5% (99% CI 16.3%, 41.9%) step increase in the percentage of high-dose codeine dispenses that were pain medicines but, with a significant trend decrease of −1.3% (99% CI −2.5%, −0.1%). There were significant trend increases in the number of opioids (excluding codeine) (1.4%, 99% CI 0.0%, 2.8%) and other pain medicines (2.0%, 99% CI 1.0%, 3.1%), but not in the percentage of pain medicines. Finally, there was a significant −12.3% (99% CI −19.0%, −5.1%) step decrease in the mean dispense dose of opioids (excluding codeine) following the up-scheduling of low-dose codeine.

**Table 3:** Results of interrupted time series

	Monthly mean (SD) values		Step change	Trend change
	2 years before*	1 year after†	% (99% CI)‡	% (99% CI)
N of dispenses				
Up-scheduled low-dose codeine	55.5 (8.7)	58.8 (12.5)	−7.9% (−29.4%, 19.5%)	2.3% (−0.9%, 5.6%)
High-dose codeine	259.8 (36.4)	265.5 (31.7)	32.3% (17.4%, 49.0%)§	0.0% (−1.4%, 1.5%)
Opioids (excl. codeine)	948.5 (89.6)	924.7 (60.7)	1.1% (−9.9%, 13.5%)	1.4% (0.0%, 2.8%)§
Other pain medicine	1213.4 (118.1)	1188.8 (89.9)	−3.6% (−12.1%, 5.9%)	2.0% (1.0%, 3.1%)§
% of pain medicines dispensed				
Up-scheduled low-dose codeine	2.2 (0.4)	2.4 (0.4)	−13.0% (−32.8%, 12.7%)	1.0% (−2.1%, 4.2%)
High-dose codeine	10.5 (0.8)	10.9 (1.0)	28.5% (16.3%, 41.9%)§	−1.3% (−2.5%, −0.2%)§
Opioids (excl. codeine)	38.3 (1.6)	38.0 (0.8)	−4.9% (−10.9%, 1.5%)	0.2% (−0.6%, 1.0%)
Other pain medicine	49.0 (1.3)	48.8 (0.7)	−4.4% (−9.6%, 1.1%)	0.3% (−0.8%, 1.3%)
Mean dispense dose (MME)				
Up-scheduled low-dose codeine	58.4 (3.7)	55.2 (4.1)	−18.5% (−27.7%, −8.0%)§	2.2% (0.8%, 3.6%)§
High-dose codeine	167.2 (6.8)	155.3 (9.3)	−8.9% (−18.0%, 1.2%)	0.6% (−0.7%, 1.9%)
Opioids (excl. codeine)	529.2 (31.6)	519.1 (25.5)	−12.3% (−19.0%, −5.1%)§	0.0% (−1.0%, 1.0%)

* 2 years/24 months before the up-scheduling of codeine (February 2016 to January 2018).

† 1 year/12 months after the up-scheduling of codeine (February 2018 to February 2019).

‡ Percentage change (99% CI).

§ p<0.01.

## Discussion

The up-scheduling of low-dose codeine did not significantly change the prevalence, monthly number or monthly percentage of low-dose codeine-containing products dispensed to injured Australian workers with workers’ compensation claims for musculoskeletal conditions. Less than 3% of workers were ever dispensed up-scheduled low-dose codeine compared with the nearly third ever dispensed other types of opioids. There was a significant step increase in the monthly number and percentage of high-dose codeine dispenses at the time of low-dose codeine up-scheduling. This was accompanied by a significant decreasing trend over the subsequent year, and high-dose codeine was significantly less prevalent in workers whose claims commenced every year after 2010. Opioids other than codeine were significantly more prevalent in those workers whose claims commenced after up-scheduling. However, there was also a significant step decrease in the monthly mean dose per opioid dispensed at the time of up-scheduling.

We did not observe the same significant decreases in low-dose codeine dispenses following up-scheduling in our sample as in other Australian studies.^8^,^1125^ This lack of change in our sample could be related to several factors. First, up-scheduled low-dose codeine products were available over the counter for many years at a relatively low cost, so it is possible that few injured workers claimed reimbursement from the workers’ compensation scheme. Second, while claims for prescription medicines are lodged by pharmacists with detailed medicine recording forms, over-the-counter medicines are not necessarily recorded in detail and are often reported as ‘over-the-counter medicine’; moving to a prescription-only medicine may have improved data quality. Finally, we included a sample of specific musculoskeletal conditions as opposed to an entire population. Our sample also represents workers with at least 2 weeks of absence from work who likely have more severe injuries.

Our findings suggest that the up-scheduling of low-dose codeine may have shifted workers already being dispensed low-dose codeine to higher doses of codeine and workers commencing claims after the up-scheduling of codeine to other opioids and other pain medicines. The potential for these shifts was raised as a concern by consumers and healthcare providers prior to up-scheduling.^26^ First, there was a significant, but temporary, increase in high-dose codeine dispenses that were ultimately less prevalent in more recent claims. Some individuals may have been purchasing numerous packs of low-dose codeine medicines per week out of pocket, which would not be detected in our data. It is unlikely that these workers would have attended a doctor multiple times per week for multiple prescriptions and may have been prescribed larger dispenses. This may be one reason behind the temporary significant increase in high-dose codeine dispenses and seems to point to workers who may have been seeking low-dose codeine prior to up-scheduling to be prescribed high-dose codeine or lower doses of other opioids if they are required to obtain a prescription either way. Workers who commenced their claim after low-dose codeine up-scheduling appear to have avoided low- and high-dose codeine products, with significant monthly trend increases and a greater prevalence of workers dispensed opioids and other pain medicines; the latter of which aligns with the findings of Schaffer *et al*.^8^

### Implications for policy and practice

Our findings indicate that a consistent proportion of workers with workers’ compensation time loss claims for musculoskeletal conditions continue to be prescribed pain medicines. This is troubling considering the negative disability, health and cost outcomes associated with opioids specifically for compensated workers who likely have long-term pain problems.^27^,^30^ The changes and trends highlighted in our study indicate that these workers have progressively shifted to a more diverse array of opioids and other pain medicines. The Victoria workers’ compensation authority has a clinical panel that conducts internal reviews of healthcare and pharmaceutical treatment of injured workers. However, it is unclear what relation the workers’ compensation scheme has with the mandatory prescription-drug monitoring programme (PDMP) that was implemented in April 2020 in Victoria (the state of this study).^31^ Workers’ compensation schemes could consider their own systems to flag certain types and doses of medicines dispensed to injured workers to supplement a PDMP if they have not already. As evidenced by our analysis, the raw data exists to achieve this.

### Strengths, limitations and future research

Our study benefited from a large sample of detailed data, enabling us to gain new insights into the impact of a critical policy change. Detailed medicines data linked with detailed worker data allowed us to adjust for numerous important covariates. Limitations of our study should be considered. First, employers must fund the first $700 (AUD) of medical expenses in the Victorian workers’ compensation scheme,^16^ which is not recorded in the administrative data. Pain medicines are relatively inexpensive in Australia, and so it is likely that we are missing at least some medicines. Second, we only included medicines funded by the workers’ compensation scheme. It is possible that over-the-counter products like low-dose codeine were purchased out-of-pocket and subsequently not detected in our data. Thirdly, background trends in medicines dispensed may have confounded interrupted time-series analyses. For example, there was an apparent rise in the use of tapentadol in the years before and after the up-scheduling of codeine that may have contributed to observed changes in the mean monthly dose of opioids. Finally, we only included a follow-up period of 1 year since the insurer received the claim, missing potentially longer-term trends in pain medicine dispensing. Workers are eligible for healthcare funding for up to 52 weeks after income replacement ends at 130 weeks in the Victorian workers’ compensation scheme. Future research could, therefore, consider follow-up periods of up to 3.5 years.^16^

### Conclusions

Up-scheduling codeine did not significantly change the dispensing of low-dose codeine-containing products to Australian workers with accepted workers’ compensation time loss claims for musculoskeletal conditions. A temporary increase in high-dose codeine, step decrease in mean opioid dispense dose and trend increases in other opioids and other pain medicines appear to indicate a shift away from any dose of codeine to lower dose opioids and other analgesics, such as pregabalin. Workers’ compensation schemes could consider utilising detailed medicine data to monitor medicine dispenses as a supplement to prescription drug monitoring programmes.

## supplementary material

10.1136/bmjopen-2024-092651 online supplemental file 1

## Data Availability

Data may be obtained from a third party and are not publicly available.
