# Supplementary material for: Trends in opioid dispensing to injured workers following codeine scheduling changes in Australia: a retrospective cohort study
Source: BMJ Open. 2025 Mar 12;15(3):e092651. doi: 10.1136/bmjopen-2024-092651 (PMC11904360; doi:10.1136/bmjopen-2024-092651)
Supplement: online supplemental file 1 [file bmjopen-15-3-s001.pdf]

Trends in opioid dispensing to injured workers following codeine scheduling changes in Australia: a retrospective cohort study

Michael Di Donato[1], Stephanie Mathieson[2], Giovanni E Ferreira[2,3], Ting Xia[4], Yonas Getaye Tefera[1], Christina Abdel Shaheed[2,3,5], Christopher G Maher[2,3] & Alex Collie[1]

1. Healthy Working Lives Research Group, School of Public Health and Preventive Medicine, Monash University
2. Sydney Musculoskeletal Health, Faculty of Medicine and Health, The University of Sydney
3. Institute for Musculoskeletal Health, Sydney Local Health District and University of Sydney
4. Monash Addiction Research Centre, Eastern Clinical School, Monash University
5. Sydney School of Public Health, Faculty of Medicine and Health, University of Sydney

Corresponding author:

Dr Michael Di Donato

Healthy Working Lives Research Group, School of Public Health and Preventive Medicine, Monash University

553 St Kilda Road, Melbourne, VIC 3004, Australia

michael.didonato@monash.edu / +61 03 9905 6417

SUPPLEMENTARY INFORMATION

Supplementary Table 1. Included musculoskeletal conditions

TOOCS	***ATC Description***
Nature of Injury	F – Traumatic Joint / Ligament and Muscle / Tendon Injury<br>H – Musculoskeletal and Connective Tissue Diseases
Bodily Location of Injury	Any
Mechanism of Injury	Any
Agency of Injury	Any

Supplementary Table 2. Anatomical Therapeutic Chemical (ATC) codes to define pain medicines

ATC Code	***ATC Description***
M01	Antiinflammatory and antirheumatic products
M02	Topical products for joint and muscular pain
M03	Muscle relaxants
N01	Anesthetics
N02	Analgesics
N03	Antiepileptics
N05	Psychopleptics
N06	Psychoanaleptics

Supplementary Table 3. Included categories of pain medicines

Pain medicine	***ATC code[1]***	***Item strength***
Up-scheduled low-dose codeine	N02AA59, N02AJ06, N02AJ07, N02AJ08, R05DA04	<= 15mg of codeine
High-dose codeine	N02AA59, N02AJ06, R05DA04	>15mg of codeine
Opioids (excl. codeine)	N02AA01, N02AA03, N02AA05, N02AA55, N02AB02, N02AB03, N02AC04, N02AC54, N02AE01, N02AJ13, N02AX02, N02AX06	Any
Other pain medicines[2]	M01, M02, M03, N01, N03, N05, N06	Any

1: Listed ATC codes represent medicines available in sample of data.

2: Other pain medicines include pregabalin, antidepressants, paracetamol, with the most frequent medicines listed in the supplementary materials.

Supplementary Table 4. Missing data report

Medicines	***Complete*** *N (%)*	***Missing*** *N (%)*
Complete analyses		
Up-scheduled low-dose codeine	7,859 (99.8%)	13 (0.2%)
High-dose codeine	36,408 (98.2%)	679 (1.8%)
Opioids (excl. codeine)	99,859 (99.8%)	211 (0.2%)
ITS analyses		
Up-scheduled low-dose codeine	2,036 (99.8%)	<5 (-)[1]
High-dose codeine	9,422 (99.5%)	45 (0.5%)
Opioids (excl. codeine)	33,861 (99.8%)	63 (0.2%)

1: Number suppressed due to small size

Supplementary Table 5. R packages used in analyses

Package	***Version***	***Reference***
MASS	7.3.58.3	https://cran.r-project.org/package=MASS
base	4.2.2	https://cran.r-project.org/package=base
broom	1.0.3	https://cran.r-project.org/package=broom
broom.mixed	0.2.9.4	https://cran.r-project.org/package=broom.mixed
datasets	4.2.2	https://cran.r-project.org/package=datasets
dplyr	1.1.3	https://cran.r-project.org/package=dplyr
forcats	1.0.0	https://cran.r-project.org/package=forcats
ggplot2	3.4.4	https://cran.r-project.org/package=ggplot2
grDevices	4.2.2	https://cran.r-project.org/package=grDevices
graphics	4.2.2	https://cran.r-project.org/package=graphics
haven	2.5.1	https://cran.r-project.org/package=haven
lubridate	1.9.1	https://cran.r-project.org/package=lubridate
methods	4.2.2	https://cran.r-project.org/package=methods
nlme	3.1.160	https://cran.r-project.org/package=nlme
patchwork	1.1.2	https://cran.r-project.org/package=patchwork
purrr	1.0.2	https://cran.r-project.org/package=purrr
readr	2.1.3	https://cran.r-project.org/package=readr
sandwich	3.0.2	https://cran.r-project.org/package=sandwich
scales	1.2.1	https://cran.r-project.org/package=scales
stats	4.2.2	https://cran.r-project.org/package=stats
stringr	1.5.1	https://cran.r-project.org/package=stringr
tibble	3.2.1	https://cran.r-project.org/package=tibble
tidyr	1.3.0	https://cran.r-project.org/package=tidyr
tidyverse	1.3.2	https://cran.r-project.org/package=tidyverse
utils	4.2.2	https://cran.r-project.org/package=utils

Supplementary Table 6. Prevalence of pain medicines by all available covariates

	Opioids and pain medicines		***Types of opioids***		
	All opioids	*Other pain medicines*	*Up-scheduled low-dose Codeine*	*High-codeine*	*Opioids (excl. codeine)*
	N (%)	*N (%)*	*N (%)*	*N (%)*	*N (%)*
All workers	22,807 (28.4)	20,790 (25.9)	2,367 (2.9)	10,358 (12.9)	18,154 (22.6)
Year insurer received claim[1]					
2010	2,751 (30.1)	2,426 (26.5)	359 (3.9)	1,530 (16.7)	1,999 (21.9)
2011	2,616 (29.0)	2,390 (26.5)	340 (3.8)	1,382 (15.3)	1,996 (22.2)
2012	2,644 (28.1)	2,356 (25.1)	313 (3.3)	1,370 (14.6)	1,978 (21.0)
2013	2,433 (27.5)	2,274 (25.7)	263 (3.0)	1,197 (13.5)	1,887 (21.4)
2014	2,475 (27.9)	2,310 (26.0)	210 (2.4)	1,194 (13.5)	1,964 (22.1)
2015	2,457 (28.4)	2,309 (26.7)	222 (2.6)	1,053 (12.2)	1,996 (23.1)
2016	2,347 (27.0)	2,202 (25.4)	228 (2.6)	909 (10.5)	1,984 (22.9)
2017	2,310 (26.4)	2,113 (24.2)	210 (2.4)	815 (9.3)	1,947 (22.3)
2018	2,774 (30.9)	2,410 (26.8)	222 (2.5)	908 (10.1)	2,403 (26.7)
Nature of Injury					
Diseases involving the synovium and related tissue	246 (19.3)	186 (14.6)	24 (1.9)	140 (11.0)	132 (10.3)
Diseases of muscle, tendon and related tissue	3,624 (30.7)	3,264 (27.7)	367 (3.1)	1,540 (13.1)	2,977 (25.2)
Joint diseases (arthropathies) and other articular	2,149 (32.1)	1,497 (22.4)	160 (2.4)	886 (13.3)	1,639 (24.5)
Other musculoskeletal and connective tissue diseases	351 (24.8)	349 (24.7)	43 (3.0)	174 (12.3)	270 (19.1)
Other soft tissue diseases	925 (27.9)	815 (24.5)	101 (3.0)	429 (12.9)	716 (21.6)
Spinal vertebrae and intervertebral disc diseases	8,279 (28.3)	8,875 (30.4)	1,046 (3.6)	4,183 (14.3)	6,574 (22.5)
Trauma to joints and ligaments	4,605 (26.2)	3,710 (21.1)	399 (2.3)	2,007 (11.4)	3,641 (20.7)
Trauma to muscles and tendons	2,628 (29.2)	2,094 (23.2)	227 (2.5)	999 (11.1)	2,205 (24.5)
Location of Injury					
Head and Neck	1,053 (29.4)	1,207 (33.7)	157 (4.4)	515 (14.4)	832 (23.3)
Lower Limbs	5,422 (26.2)	4,054 (19.6)	416 (2.0)	2,387 (11.5)	4,058 (19.6)
Multiple Locations	414 (29.0)	453 (31.7)	75 (5.2)	194 (13.6)	320 (22.4)
Trunk	7,635 (27.7)	8,069 (29.2)	948 (3.4)	3,888 (14.1)	6,058 (22.0)
Upper Limbs	8,283 (30.7)	7,007 (25.9)	771 (2.9)	3,374 (12.5)	6,886 (25.5)
Sex					
Female	7,973 (27.0)	8,064 (27.3)	1,240 (4.2)	3,866 (13.1)	6,078 (20.6)
Male	14,834 (29.2)	12,726 (25.1)	1,127 (2.2)	6,492 (12.8)	12,076 (23.8)
Age Group					
15-24 years	981 (15.5)	860 (13.6)	80 (1.3)	405 (6.4)	775 (12.2)

25-34 years	3,388 (23.6)	3,211 (22.4)	371 (2.6)	1,580 (11.0)	2,726 (19.0)
35-44 years	5,419 (30.0)	5,080 (28.1)	646 (3.6)	2,649 (14.6)	4,290 (23.7)
45-54 years	7,301 (31.2)	6,653 (28.4)	779 (3.3)	3,417 (14.6)	5,767 (24.7)
55-64 years	5,206 (31.9)	4,567 (27.9)	449 (2.7)	2,121 (13.0)	4,179 (25.6)
65 or more years	512 (28.3)	419 (23.1)	42 (2.3)	186 (10.3)	417 (23.0)
Employment Type					
Casual worker	580 (22.9)	537 (21.2)	39 (1.5)	219 (8.7)	493 (19.5)
Full time employee	16,371 (30.1)	14,687 (27.0)	1,542 (2.8)	7,300 (13.4)	13,114 (24.1)
Others	2,280 (23.5)	2,061 (21.3)	232 (2.4)	1,128 (11.6)	1,779 (18.4)
Part time employee	3,576 (26.0)	3,505 (25.5)	554 (4.0)	1,711 (12.5)	2,768 (20.2)
Employer Size					
Government	1,011 (27.1)	960 (25.8)	134 (3.6)	480 (12.9)	780 (20.9)
Large	6,228 (28.9)	5,809 (27.0)	758 (3.5)	2,870 (13.3)	4,852 (22.5)
Medium	8,938 (28.0)	8,185 (25.6)	890 (2.8)	4,108 (12.9)	7,128 (22.3)
Small	5,549 (28.5)	4,864 (25.0)	464 (2.4)	2,456 (12.6)	4,537 (23.3)
Missing	1,081 (29.6)	972 (26.7)	121 (3.3)	444 (12.2)	857 (23.5)
Occupation					
Advanced clerical and service workers	261 (28.8)	252 (27.8)	36 (4.0)	123 (13.6)	203 (22.4)
Associate Professionals	2,164 (28.8)	1,987 (26.5)	268 (3.6)	970 (12.9)	1,693 (22.6)
Elementary clerical, sales and service workers	900 (27.3)	864 (26.2)	118 (3.6)	421 (12.8)	717 (21.7)
Intermediate clerical, sales and service workers	2,800 (27.0)	2,731 (26.3)	401 (3.9)	1,351 (13.0)	2,161 (20.8)
Intermediate production and transport workers	4,540 (29.7)	4,032 (26.4)	375 (2.5)	2,119 (13.9)	3,603 (23.6)
Labourers and related workers	4,736 (27.7)	4,277 (25.0)	413 (2.4)	2,200 (12.8)	3,811 (22.3)
Managers and administrators	807 (33.8)	694 (29.0)	76 (3.2)	328 (13.7)	662 (27.7)
Professionals	2,447 (27.7)	2,405 (27.3)	373 (4.2)	1,103 (12.5)	1,919 (21.8)
Tradespersons and related workers	4,152 (28.4)	3,548 (24.3)	307 (2.1)	1,743 (11.9)	3,385 (23.2)
Socioeconomic Status					
Fifth quintile	3,655 (26.2)	3,448 (24.7)	399 (2.9)	1,527 (10.9)	2,884 (20.6)
Middle three quintiles	15,391 (28.8)	13,891 (26.0)	1,577 (3.0)	6,980 (13.1)	12,298 (23.0)
First quintile	3,710 (29.0)	3,409 (26.6)	386 (3.0)	1,822 (14.2)	2,930 (22.9)
Missing (n=suppressed)	51 (37.0)	42 (30.4)	-	-	-
Remoteness					
Major Cities	16,628 (28.9)	15,317 (26.6)	1,722 (3.0)	7,446 (12.9)	13,117 (22.8)
Inner Regional	5,121 (27.0)	4,558 (24.1)	531 (2.8)	2,402 (12.7)	4,161 (22.0)
Outer Regional and Remote	1,036 (27.5)	897 (23.8)	111 (2.9)	498 (13.2)	858 (22.8)
Missing (n=supressed)	22 (37.9)	18 (31.0)	-	-	-

1: Year that the insurer received the claim, where each year commenced 1st February and ended 31st January

Supplementary Table 7. Statistical comparison of the prevalence of all opioids and other pain medicines

	Opioids and pain medicines				***Types of opioids***					
	All Opioids (incl. codeine)		*Other pain medicines*		*Up-scheduled low-dose codeine*		*High-dose codeine*		*Opioids (excl. codeine)*	
	PR (99%CI)[2]	*p*	*PR (99%CI)[2]*	*p*	*PR (99%CI)[2]*	*p*	*PR (99%CI)[2]*	*p*	*PR (99%CI)[2]*	*p*
Insurer received claim[1]										
2010	1.13 (1.06, 1.20)	p<0.001	1.05 (0.98, 1.13)	0.104	1.52 (1.21, 1.92)	p<0.001	1.75 (1.57, 1.94)	p<0.001	0.97 (0.90, 1.05)	0.4
2011	1.09 (1.02, 1.16)	0.005	1.06 (0.99, 1.13)	0.066	1.48 (1.17, 1.87)	p<0.001	1.61 (1.44, 1.80)	p<0.001	0.98 (0.91, 1.06)	0.614
2012	1.04 (0.98, 1.11)	0.169	0.99 (0.93, 1.07)	0.862	1.27 (1.00, 1.61)	0.012	1.52 (1.36, 1.69)	p<0.001	0.92 (0.86, 1.00)	0.017
2013	1.03 (0.97, 1.10)	0.288	1.02 (0.96, 1.10)	0.451	1.16 (0.91, 1.49)	0.115	1.42 (1.27, 1.60)	p<0.001	0.94 (0.88, 1.02)	0.088
2014	1.04 (0.98, 1.11)	0.159	1.05 (0.98, 1.12)	0.132	0.93 (0.72, 1.21)	0.484	1.41 (1.26, 1.58)	p<0.001	0.98 (0.91, 1.06)	0.641
2015	1.07 (1.00, 1.14)	0.023	1.08 (1.01, 1.16)	0.014	1.00 (0.78, 1.30)	0.961	1.29 (1.14, 1.45)	p<0.001	1.03 (0.96, 1.11)	0.327
2016	1.01 (0.94, 1.08)	0.81	1.02 (0.95, 1.10)	0.454	1.05 (0.82, 1.36)	0.591	1.10 (0.97, 1.24)	0.062	1.01 (0.94, 1.09)	0.761
2017	1.00 (ref)	-	1.00 (ref)	-	1.00 (ref)	-	1.00 (ref)	-	1.00 (ref)	-
2018	1.18 (1.11, 1.26)	p<0.001	1.11 (1.03, 1.19)	p<0.001	1.05 (0.82, 1.36)	0.589	1.09 (0.96, 1.23)	0.092	1.21 (1.13, 1.31)	p<0.001
Nature of Injury										
Diseases involving the synovium and related tissue	0.67 (0.57, 0.78)	p<0.001	0.58 (0.48, 0.69)	p<0.001	0.60 (0.35, 1.04)	0.018	0.88 (0.71, 1.09)	0.153	0.44 (0.35, 0.55)	p<0.001
Diseases of muscle, tendon and related tissue	1.02 (0.96, 1.07)	0.508	1.10 (1.04, 1.16)	p<0.001	1.03 (0.84, 1.27)	0.689	0.98 (0.89, 1.07)	0.563	1.03 (0.97, 1.09)	0.341
Joint diseases (arthropathies)	1.19 (1.12, 1.26)	p<0.001	1.05 (0.97, 1.12)	0.158	1.11 (0.87, 1.42)	0.28	1.11 (1.01, 1.22)	0.012	1.17 (1.09, 1.25)	p<0.001
Other musculoskeletal and connective tissue diseases	0.96 (0.84, 1.10)	0.537	1.06 (0.92, 1.21)	0.372	1.02 (0.64, 1.62)	0.901	1.05 (0.85, 1.30)	0.596	0.95 (0.81, 1.11)	0.434
Other soft tissue diseases	1.00 (0.92, 1.08)	0.911	1.07 (0.98, 1.17)	0.096	1.09 (0.81, 1.45)	0.469	1.01 (0.89, 1.15)	0.84	0.99 (0.90, 1.08)	0.741
Spinal vertebrae and intervertebral disc diseases	1.26 (1.14, 1.40)	p<0.001	1.36 (1.23, 1.51)	p<0.001	1.07 (0.77, 1.49)	0.593	1.21 (1.04, 1.42)	0.004	1.31 (1.16, 1.47)	p<0.001
Trauma to joints and ligaments	1.00 (ref)	-	1.00 (ref)	-	1.00 (ref)	-	1.00 (ref)	-	1.00 (ref)	-
Trauma to muscles and tendons	1.03 (0.97, 1.09)	0.302	1.00 (0.94, 1.07)	0.923	1.00 (0.80, 1.25)	0.994	0.91 (0.82, 1.00)	0.014	1.07 (1.00, 1.14)	0.017
Location of Injury										
Upper Limbs	1.00 (ref)	-	1.00 (ref)	-	1.00 (ref)	-	1.00 (ref)	-	1.00 (ref)	-
Lower Limbs	0.83 (0.79, 0.86)	p<0.001	0.77 (0.73, 0.81)	p<0.001	0.71 (0.60, 0.85)	p<0.001	0.91 (0.85, 0.98)	0.002	0.74 (0.70, 0.78)	p<0.001
Trunk	0.76 (0.69, 0.84)	p<0.001	0.90 (0.82, 1.00)	0.022	1.15 (0.84, 1.59)	0.239	0.94 (0.81, 1.10)	0.356	0.71 (0.63, 0.80)	p<0.001
Head and Neck	0.81 (0.72, 0.90)	p<0.001	1.02 (0.91, 1.14)	0.708	1.34 (0.94, 1.93)	0.029	0.96 (0.80, 1.14)	0.548	0.75 (0.66, 0.86)	p<0.001

Multiple Locations	0.88 (0.78, 0.99)	0.018	1.09 (0.97, 1.23)	0.092	1.59 (1.14, 2.22)	p<0.001	0.97 (0.80, 1.18)	0.709	0.82 (0.71, 0.95)	0.002
Sex										
Male	1.00 (ref)	-	1.00 (ref)	-	1.00 (ref)	-	1.00 (ref)	-	1.00 (ref)	-
Female	0.94 (0.91, 0.98)	0.002	1.10 (1.06, 1.15)	p<0.001	1.61 (1.40, 1.86)	p<0.001	1.05 (0.99, 1.12)	0.066	0.88 (0.84, 0.92)	p<0.001
Age Group										
15-24 years	0.51 (0.47, 0.56)	p<0.001	0.49 (0.45, 0.54)	p<0.001	0.43 (0.31, 0.58)	p<0.001	0.44 (0.38, 0.51)	p<0.001	0.51 (0.46, 0.56)	p<0.001
25-34 years	0.75 (0.72, 0.79)	p<0.001	0.77 (0.73, 0.81)	p<0.001	0.83 (0.70, 0.98)	0.004	0.75 (0.69, 0.81)	p<0.001	0.76 (0.72, 0.81)	p<0.001
35-44 years	0.95 (0.91, 0.98)	0.002	0.97 (0.93, 1.01)	0.112	1.11 (0.97, 1.28)	0.054	0.97 (0.91, 1.04)	0.298	0.94 (0.90, 0.99)	0.006
45-54 years	1.00 (ref)	-	1.00 (ref)	-	1.00 (ref)	-	1.00 (ref)	-	1.00 (ref)	-
55-64 years	1.01 (0.97, 1.05)	0.534	1.00 (0.95, 1.04)	0.833	0.88 (0.75, 1.03)	0.036	0.90 (0.84, 0.96)	p<0.001	1.02 (0.98, 1.07)	0.301
65 or more years	0.89 (0.81, 0.99)	0.015	0.85 (0.76, 0.95)	0.001	0.82 (0.54, 1.23)	0.216	0.75 (0.62, 0.90)	p<0.001	0.90 (0.80, 1.01)	0.037
Employment Type										
Full time employee	1.00 (ref)	-	1.00 (ref)	-	1.00 (ref)	-	1.00 (ref)	-	1.00 (ref)	-
Part time employee	0.87 (0.83, 0.92)	p<0.001	0.86 (0.82, 0.91)	p<0.001	1.00 (0.86, 1.16)	0.936	0.90 (0.83, 0.97)	p<0.001	0.87 (0.82, 0.92)	p<0.001
Others	0.89 (0.84, 0.93)	p<0.001	0.88 (0.84, 0.93)	p<0.001	0.91 (0.76, 1.10)	0.213	0.92 (0.85, 1.00)	0.017	0.88 (0.82, 0.93)	p<0.001
Casual worker	0.83 (0.75, 0.92)	p<0.001	0.86 (0.77, 0.95)	p<0.001	0.68 (0.44, 1.04)	0.021	0.91 (0.76, 1.09)	0.184	0.82 (0.73, 0.92)	p<0.001
Employer Size										
Small	1.04 (1.00, 1.08)	0.032	1.03 (0.99, 1.07)	0.126	0.98 (0.85, 1.14)	0.74	1.02 (0.96, 1.09)	0.398	1.06 (1.01, 1.10)	0.005
Medium	1.00 (ref)	-	1.00 (ref)	-	1.00 (ref)	-	1.00 (ref)	-	1.00 (ref)	-
Large	1.03 (0.99, 1.07)	0.077	1.01 (0.97, 1.05)	0.454	1.05 (0.92, 1.20)	0.317	1.03 (0.97, 1.09)	0.298	1.02 (0.97, 1.06)	0.386
Government	0.94 (0.87, 1.02)	0.104	0.95 (0.87, 1.03)	0.132	0.94 (0.73, 1.20)	0.503	1.00 (0.88, 1.13)	0.949	0.92 (0.84, 1.01)	0.046
Occupation										
Labourers and related workers	1.00 (ref)	-	1.00 (ref)	-	1.00 (ref)	-	1.00 (ref)	-	1.00 (ref)	-
Intermediate production and transport workers	1.00 (0.95, 1.05)	0.948	1.00 (0.95, 1.05)	0.911	1.08 (0.89, 1.30)	0.32	1.02 (0.95, 1.10)	0.524	0.98 (0.92, 1.03)	0.306
Tradespersons and related workers	1.02 (0.97, 1.07)	0.346	1.01 (0.96, 1.06)	0.699	1.05 (0.86, 1.29)	0.519	0.97 (0.89, 1.05)	0.295	1.02 (0.97, 1.08)	0.342
Intermediate clerical, sales and service workers	1.02 (0.96, 1.08)	0.523	1.02 (0.96, 1.08)	0.52	1.25 (1.03, 1.52)	0.003	0.99 (0.91, 1.09)	0.874	1.02 (0.96, 1.09)	0.487
Associate Professionals	1.05 (0.99, 1.13)	0.085	1.03 (0.96, 1.11)	0.314	1.30 (1.03, 1.64)	0.004	1.01 (0.91, 1.13)	0.792	1.04 (0.96, 1.12)	0.277
Professionals	1.05 (0.99, 1.12)	0.079	1.05 (0.99, 1.12)	0.072	1.37 (1.12, 1.68)	p<0.001	0.95 (0.86, 1.05)	0.244	1.08 (1.00, 1.16)	0.016
Elementary clerical, sales and service workers	1.02 (0.94, 1.11)	0.567	1.03 (0.95, 1.12)	0.38	1.26 (0.96, 1.66)	0.027	0.98 (0.86, 1.12)	0.739	1.05 (0.96, 1.15)	0.222

Managers and administrators	1.16 (1.07, 1.26)	p<0.001	1.10 (1.00, 1.20)	0.028	1.24 (0.90, 1.71)	0.092	1.03 (0.89, 1.18)	0.678	1.19 (1.08, 1.30)	p<0.001
Advanced clerical and service workers	1.05 (0.91, 1.21)	0.458	1.04 (0.90, 1.21)	0.527	1.28 (0.81, 2.01)	0.178	1.00 (0.80, 1.26)	0.986	1.08 (0.91, 1.27)	0.327
Socioeconomic Status										
Fifth quintile	0.90 (0.86, 0.94)	p<0.001	0.93 (0.89, 0.97)	p<0.001	0.90 (0.77, 1.04)	0.068	0.83 (0.77, 0.89)	p<0.001	0.90 (0.86, 0.95)	p<0.001
Middle three quintiles	1.00 (ref)	-	1.00 (ref)	-	1.00 (ref)	-	1.00 (ref)	-	1.00 (ref)	-
First quintile	1.03 (0.99, 1.07)	0.142	1.05 (1.01, 1.10)	0.008	1.09 (0.94, 1.26)	0.15	1.10 (1.03, 1.18)	p<0.001	1.01 (0.96, 1.05)	0.81
Remoteness										
Major Cities	1.00 (ref)	-	1.00 (ref)	-	1.00 (ref)	-	1.00 (ref)	-	1.00 (ref)	-
Inner Regional	0.92 (0.89, 0.95)	p<0.001	0.89 (0.86, 0.93)	p<0.001	0.92 (0.81, 1.05)	0.118	0.94 (0.88, 1.00)	0.01	0.95 (0.91, 1.00)	0.013
Outer Regional and Remote	0.92 (0.85, 0.99)	0.01	0.87 (0.80, 0.94)	p<0.001	0.97 (0.75, 1.26)	0.773	0.93 (0.83, 1.05)	0.144	0.97 (0.89, 1.06)	0.42

1: Year that the insurer received the claim, where each year commenced 1st February and ended 31st January

2: Prevalence Ratio and 99% Confidence Interval

3: Poisson model adjusted for worker sex, age group, employment type, employer size, nature of injury, bodily location of injury, occupation, socioeconomic status and remoteness

**: $p < 0.01$*

Supplementary Table 8. Summary of top five most frequent opioids and other pain medicines before and after up-scheduling

No.	***Two years before up-scheduling***	***N (%)***	***Year after up-scheduling***	***N (%)***
Up-scheduled low-dose codeine				
1	N02AJ06 Codeine and paracetamol (15mg)	537 (40.3)	N02AJ08 Codeine and ibuprofen (12.8mg)	199 (28.1)
2	N02AJ06 Codeine and paracetamol (8mg)	291 (21.9)	N02AJ06 Codeine and paracetamol (15mg)	173 (24.5)
3	N02AJ08 Codeine and ibuprofen (12.8mg)	187 (14)	N02AA59 Codeine, combinations excl. psycholeptics (9.75mg)	157 (22.2)
4	N02AA59 Codeine, combinations excl. psycholeptics (9.75mg)	185 (13.9)	N02AJ06 Codeine and paracetamol (8mg)	90 (12.7)
5	N02AJ06 Codeine and paracetamol (10mg)	49 (3.7)	N02AJ06 Codeine and paracetamol (9.6mg)	53 (7.5)
High-dose codeine				
1	N02AJ06 Codeine and paracetamol (30mg)	5,814 (93.2)	N02AJ06 Codeine and paracetamol (30mg)	2,941 (92)
2	N02AA59 Codeine, combinations excl. psycholeptics (30mg)	411 (6.6)	N02AA59 Codeine, combinations excl. psycholeptics (30mg)	256 (8)
3	R05DA04 Codeine (30mg)	11 (0.2)	R05DA04 Codeine (30mg)	< 5 (-)
Opioids (excluding codeine)				
1	N02AA05 Oxycodone (5mg)	6,886 (30.2)	N02AA05 Oxycodone (5mg)	3,486 (31.3)
2	N02AX02 Tramadol (50mg)	2,773 (12.2)	N02AX06 Tapentadol (50mg)	1,275 (11.4)
3	N02AA55 Oxycodone and naloxone (10mg)	2,562 (11.3)	N02AX02 Tramadol (50mg)	1,265 (11.4)
4	N02AX02 Tramadol (100mg)	1,395 (6.1)	N02AA55 Oxycodone and naloxone (10mg)	1,170 (10.5)
5	N02AA55 Oxycodone and naloxone (5mg)	1,317 (5.8)	N02AA55 Oxycodone and naloxone (5mg)	587 (5.3)
Other medicines used for pain				
1	N02BF02 Pregabalin	6,420 (22.0)	N02BF02 Pregabalin	2,945 (20.6)
2	N02BE01 Paracetamol	3,149 (10.8)	N02BE01 Paracetamol	1,725 (12.0)
3	M01AC06 Meloxicam	2,437 (8.4)	M01AC06 Meloxicam	1,165 (8.1)
4	M01AH01 Celecoxib	2,085 (7.2)	M01AH01 Celecoxib	1,070 (7.5)
5	N05BA01 Diazepam	2,002 (6.9)	N06AA09 Amitriptyline	938 (6.5)
