# Supplementary material for: Trends in opioid dispensing to injured workers following codeine scheduling changes in Australia: a retrospective cohort study
Source: BMJ Open. 2025 Mar 12;15(3):e092651. doi: 10.1136/bmjopen-2024-092651 (PMC11904360; doi:10.1136/bmjopen-2024-092651)
Supplement: Supplementary file 2 [file bmjopen-2024-092651.reviewer_comments.pdf]

PEER REVIEW HISTORY

BMJ Open publishes all reviews undertaken for accepted manuscripts. Reviewers are asked to complete a checklist review form and are provided with free text boxes to elaborate on their assessment. These free text comments are reproduced below.

ARTICLE DETAILS

Title (Provisional)

Trends in opioid dispensing to injured workers following codeine scheduling changes in Australia: a retrospective cohort study

Authors

Di Donato, Michael F; Mathieson, Stephanie; Ferreira, Giovanni E; Xia, Ting; Tefera, Yonas Getaye; Abdel Shaheed, Christina; Maher, Chris; Collie, Alex

---

VERSION 1 - REVIEW

**Reviewer**	**1**
**Name**	**Canizares, Mayilee**
**Affiliation**	**UHN, Arthritis Program**
**Date**	**11-Nov-2024**
**COI**	**None**

The manuscript "Trends in opioid dispensing to injured workers following codeine scheduling changes in Australia: a retrospective cohort study" describes patterns of pain medication use among injured workers in Australia. The data and analytical methods used are appropriate for the study objectives. The paper is clearly layout and written. See few comments/questions below.

1. Was the use of low dose codeine before Feb 2018 through OTC only? Or there were also cases in which a prescription was issued? I'm trying to understand if the datasets used are capturing the low-dose codeine more accurately after 2018 and how this may have an impact on the results. Please clarify.
2. Page 5, lines 136-137: Are there any guidelines in Australia recommending limit dosages (e.g., <50, <90 MMEs/day)? If so, did the authors examine changes in the proportion of workers with prescriptions within these limits?
3. Page 5, line 144: Please check "Error! Reference source not found."
4. Page 11, lines 287-289: Could the authors expand on the potential factors underpinning the shift from low-dose to high-dose codeine?
5. I wonder if the patterns of pain medication use over time presented in the paper differ by any major variables like for example, the nature of injury, or se, age, occupation etc. Could you comment on this?

**Reviewer**	**2**

Name Franklin, Gary

Affiliation University of Washington, School of Public Health & Community Medicine

Date 12-Nov-2024

COI None

This is a well done paper by this prolific group. The methods are solid, and the results are clearly reported. The findings related to ongoing high prevalence of opioid use and increasing tapentadol use in workers comp are worrisome and important for future policy considerations. I don't have any critique of the methods or findings.

VERSION 1 - AUTHOR RESPONSE

Reviewer #1	
The manuscript "Trends in opioid dispensing to injured workers following codeine scheduling changes in Australia: a<br>retrospective cohort study" describes patterns of pain medication use among injured workers in Australia. The data and analytical methods used are appropriate for the study objectives. The paper is clearly layout and written. See few<br>comments/questions below.	Thank you for your review.
1. Was the use of low dose codeine before Feb 2018 through OTC only? Or there were also cases in which a prescription was issued? I'm trying to understand if the datasets used are capturing the low-dose codeine more accurately after 2018 and how this may have an impact on the results. Please clarify.	Low-dose codeine was available over-the-counter, but greater volumes (i.e., greater numbers of tablets) required a prescription.<br>We agree that data capture may be one reason, and acknowledged this in the discussion. We have made this more clear with the addition of some text (page 11, line 326).
2. Page 5, lines 136-137: Are there any guidelines in Australia recommending limit dosages (e.g., <50, <90 MMEs/day)? If so, did the authors examine changes in the proportion of workers with prescriptions within these limits?	There are various guidelines in each state and territory with relatively consistent recommendations about dose. We did not explore the proportion of workers within these prescription limits. This would be an interesting future study, but we believe it is out of scope for this paper.
3. Page 5, line 144: Please check "Error! Reference source not found."	Apologies, this has been corrected. The reference should have been to Supplementary Table 2.
4. Page 11, lines 287-289: Could the authors expand on the potential factors underpinning the shift from low-dose to high- dose codeine?	We have provided our theory on the reason for this shift in the subsequent sentences (page 11, lines 333-342). We do not wish to extend our hypotheses too far based on this observational study.

5. I wonder if the patterns of pain medication use over time presented in the paper differ by any major variables like for example, the nature of injury, or se, age, occupation etc. Could you comment on this?	This is a definite possibility. Our supplementary tables 6 and 7 provide information about pain medicines dispensing based on worker characteristics, but these are for the total sample, not over time.<br>Workers' compensation claims for musculoskeletal conditions remain relatively constant in Australia. Any changes over time may be due to the introduction of new medicines or medicine policy changes.<br>This would be an interesting topic for future study and a topic we are exploring.
Reviewer #2	
This is a well done paper by this prolific group. The methods are solid, and the results are clearly reported. The findings related to ongoing high prevalence of opioid use and increasing tapentadol use in workers comp are worrisome and important for future policy considerations. I don't have any critique of the methods or findings.	Thank you for this very positive feedback.

VERSION 2 - REVIEW

Reviewer 1

Name Canizares, Mayilee

Affiliation UHN, Arthritis Program

Date 03-Feb-2025

COI

The authors have addressed my questions. I have no further comments.
